# Does Pioglitazone Lead to Neutrophil Extracellular Traps Formation in Chronic Granulomatous Disease Patients?

**DOI:** 10.3389/fimmu.2019.01739

**Published:** 2019-07-31

**Authors:** Gouri P. Hule, Umair Ahmed Bargir, Manasi Kulkarni, Priyanka Kambli, Prasad Taur, Mukesh Desai, Manisha Rajan Madkaikar

**Affiliations:** ^1^Department of Paediatric Immunology and Leukocyte Biology, National Institute of Immunohaematology (ICMR), Mumbai, India; ^2^Bai Jerbai Wadia Hospital for Children, Mumbai, India

**Keywords:** peroxisome proliferator-activated receptor gamma agonists, pioglitazone, chronic granulomatous disease, neutrophil extracellular traps, reactive oxygen species, mitochondrial ROS, MitoSOX red, NOX independnent NETosis

## Abstract

Nicotinamide adenine dinucleotide phosphate (NADPH) oxidase, the enzyme complex responsible for reactive oxygen species (ROS) production, is defective in chronic granulomatous disease (CGD) patients. This enzyme helps in antimicrobial host defense by phagocytes. CGD patients are unable to form neutrophil extracellular traps (NETs), which are composed of granule-derived proteins from neutrophils decorated with decondensed chromatin. Mitochondria have gained attention, being a rich source of flavochrome enzymes due to the presence of several sites for superoxide production. Recently, PPARγ agonists, a mitochondrial ROS inducer, induce mitochondrial ROS formation post-treatment in murine NADPH oxidase knockout models. Mitochondrial ROS is also essential for NOX-independent NETosis. Our study for the first time detects induction of NETosis independent of NADPH oxidase post-treatment with agonists such as pioglitazone and rosiglitazone in CGD subjects. Neutrophils isolated from CGD subjects were treated with pioglitazone and rosiglitazone. After treatment, qualitative analysis of NET formation was done using confocal microscopy after staining with DAPI. Quantitative estimation of extracellular DNA was performed using Sytox green. Mitochondrial ROS production with PPARγ agonist-treated/untreated neutrophils was detected using MitoSOX red. Pioglitazone and rosiglitazone induce significant NET formation in CGD patients. Our data clearly signify the effect of PPARγ agonists in induction of NET formation in CGD cases. Apart from the proposed experimental studies regarding the detailed mechanism of action, controlled trials could provide valuable information regarding the clinical use of pioglitazone in CGD patients as curative HSCT remains challenging in developing countries.

## Introduction

Nicotinamide adenine dinucleotide phosphate (NADPH) oxidase is a crucial enzyme in antimicrobial host defense by phagocytes and also plays an important role in regulating inflammation. Genetic defect in NADPH oxidase (NOX2) enzyme components impairs formation of reactive oxygen species (ROS). Apart from NADPH oxidase, other known sources of ROS producers are xanthine oxidase, nitric oxide synthase, cytochrome P450, and mitochondrial electron transport chain (ETC). Among these ROS producers, mitochondria have drawn increasing attention as they are a rich source of flavochrome enzymes and have several sites for superoxide production (1–3). Several inducers of mitochondrial ROS such as oxidized low-density lipoproteins, glucose, tumor necrosis factor alpha, angiotensin, and peroxisome proliferator-activated receptor gamma (PPARγ) agonists are known (4). PPARγ agonists have been shown to affect mitochondrial respiratory chain functions (5, 6). Also, PPARγ agonists pioglitazone and rosiglitazone are used for treatment of type II diabetes patients (7, 8). Pioglitazone selectively stimulates the nuclear receptor PPAR-γ and modulates the transcription of the genes involved in control of glucose and lipid metabolism in muscle, liver, and adipose tissues (9). Pioglitazone helps in production of interleukin-10, thereby suppressing systemic inflammatory cytokine production (10). On the other hand, rosiglitazone was also found to modulate the inflammatory response and increase bacterial clearance through PPARγ activation (11). Recent study shows the induction of mitochondrial ROS production and restored killing of *Staphylococcus aureus* and *Burkholderia cepacia*, post-pioglitazone treatment in murine NADPH oxidase knockout models (12). Baseline mitochondrial ROS production was also detected in NADPH oxidase defective (Chronic granulomatous disease) patients (13).

Chronic granulomatous disease (CGD) patients have impaired neutrophil extracellular traps (NETs) formation due to absence of NADPH oxidase generated ROS molecules. NETs are composed of chromatin associated with an array of granule-derived proteins such as neutrophil elastase (NE), myeloperoxidase (MPO), histones, and proteolytic enzymes (14, 15). These NETs traps bacteria, fungus, and protozoa (15–17), thereby creating an antimicrobial proteins milieu. Various stimuli such as phorbol 12-myristate 13-acetate (PMA), micro-organisms, calcium ionomycin, uric acid, and cytokines/chemokines are known to induce NET formation (NETosis) (18–22). NADPH oxidase-dependent NETosis is stimulated by a potent mitogen, PMA, via protein kinase C (PKC) activation (15). Calcium ionomycin and uric acid induce NETosis-independent of ROS generated by NADPH oxidase. A recent study states that mitochondrial ROS is required for NET formation independent of NADPH oxidase (23). Earlier studies clearly state that NETosis is generated independent of ROS generated by NADPH oxidases and also involves mitochondrial ROS in some cases. This study hypothesizes whether NETosis is generated independent of NADPH oxidase after treatment with pioglitazone in CGD cases with varying germline mutations.

We studied for the first time the induction of NETosis by PPARγ agonists such as pioglitazone in CGD subjects. Additionally, mitochondrial ROS production post-PPARγ agonist treatment was also studied in CGD subjects with varying germline mutations.

## Materials and Methods

### Subject Selection

The study was conducted under ethics approval from Bai Jerbai Wadia Hospital for children (reference number: IEC-BJWHC/AP/2017/012). Written informed parental consent was obtained. The study included five CGD patients diagnosed at our center. Peripheral blood was collected in EDTA blood collection tubes. Age-matched control cohort was also incorporated in the study.

### Diagnosis of CGD Cases

A defect in the NADPH oxidase enzyme complex was detected by NBT dye reduction test and DHR oxidation assays. Absence of NBT dye reduction and oxidation of DHR dye upon stimulation with PMA (29 nM) is indicative of defective NADPH oxidase enzyme. The protocol for NBT and DHR was followed as mentioned (24). Expression of the NADPH component was evaluated using monoclonal antibodies against p22^phox^ (clone sc-130550, Santa Cruz biotechnology), p47^phox^ (clone 1, BD Biosciences), and p67^phox^ (clone D-6, Santa Cruz Biotechnology) in a flow-cytometry-based approach. The stain-lyse-wash protocol was used to study the NADPH oxidase enzyme complex components such as p22^phox^, p47^phox^, and p67^phox^. The expression of components was evaluated on neutrophils. These components were evaluated using a described permeabilization protocol (24). A minimum of 10,000 neutrophils per tube were acquired on BD FACS Aria Fusion (special order system) using FACS-Diva software, and data were further analyzed using FlowJo software (Tree Star Inc.).

### Molecular Characterization of Diagnosed CGD Cases

Genomic DNA was isolated from peripheral blood of patients collected in EDTA tubes using the whole blood DNA extraction kit (Qiagen). All the exons, along with the intron–exon boundaries of *CYBB, NCF2*, and *CYBA* genes were amplified by polymerase chain reaction (PCR) and were run on 1.5% agarose gel. The PCR products were sequenced by Sanger sequencing (performed at NIIH, ABI 3130 Xl genetic analyser, Applied Biosystems) and the results were analyzed by the BLAST program. In case of *NCF1* gene analysis, the GeneScan (GeneMapper™ Software, Thermo Fisher Scientific) assay was performed to calculate the ratio of pseudo *NCF1* gene to *NCF1* gene (24).

### Isolation of Neutrophil

Isolation protocol devoid of dextran sedimentation, multi-step centrifugation, and without use of any type of lysing solution was selected, to avoid activation of neutrophils. Isolation of neutrophils (>95% pure) from healthy and CGD patients was performed using discontinuous Percoll (Sigma Aldrich) gradients as described (25).

### Treatment of Neutrophils for Inducing NET Formation

Sterile round coverslips were placed inside 12-well sterile Nunclon delta surface (Thermo Scientific) culture plates. Coverslips were coated with 0.001% poly L-lysine (Sigma Aldrich) for 30 min and neutrophils (1 × 10^5^ cells) were loaded after removal of coating solution. Neutrophils from patient/control were subjected to stimulation with or without [PPARγ antagonists, GW9662 (Sigma; 10 μg/μl)] along with stimulation by PPARγ agonists pioglitazone (14 μg/μl; Sigma) and rosiglitazone (15 μg/μl; Sigma) for 18–20 h at 37°C in a CO_2_ incubator. Positive control: neutrophils were stimulated with PMA for 4 h at 37°C in a CO_2_ incubator. Negative controls: cells were not treated with any stimulant.

After treatment, cells were fixed with 4% paraformaldehyde (PFA). Treatment with detergent (0.1% Triton X) and blocking was done using 1% bovine serum albumin (BSA) at room temperature. After blocking, cells were washed and subsequently were stained with 4′,6-diamidino-2-phenylindole (DAPI; Sigma Aldrich) and with anti-human myeloperoxidase (MPO) antibody tagged to fluorescein isothiocyanate (FITC) (1:50, Becton Dickinson). Negative control samples [unstimulated/treated with dimethyl sulfoxide (DMSO)] were processed similarly as mentioned above, omitting the stimulant step. NETs were assessed by observing NETs forming neutrophils using confocal microscopy (Carl Zeiss LSM 510 META) under 63× (26).

### Quantitation of NET Formation

After neutrophil treatment step, NETs-bound DNA was quantified using Sytox green (5 μM, Invitrogen) and fluorescence was measured at 504 nm (excitation) and 530 nm (emission) using Tecan Infinite M200 Pro (Switzerland).

### Quantitation of Mitochondria ROS by MitoSOX Red

Mitochondrial ROS was quantitated by MitoSOX red (4 mmol/L; Life Technologies; for 15 min only) with or without the treatment of neutrophils with mitochondrial ROS inhibitor MitoTempo (100 mmol/L; Sigma) for 30 min followed by agonist stimulation and fluorescence was measured at 510 nm (excitation) and 580 nm (emission) using Tecan Infinite M200 Pro (Switzerland).

### Statistics

Data are presented as mean ± SD and analyzed using two-sided Student's *t*-test. Analysis was performed using GraphPad prism (version 7). *P* < 0.05 was considered statistically significant.

## Results

### Clinical Characteristics and Cellular ROS Production in CGD Subjects

Clinical details and functional parameters for CGD cases involved in this study are documented in Table 1. Details include age of diagnosis (in months), total leukocyte count (TLC), absolute neutrophil count (ANC), and absolute lymphocyte count (ALC). Majority of patients had leucocytosis (4 out of 5), pneumonia (4 out of 5), and skin abscesses (3 out of 5) with lung being a common site of infection (3 out of 5). Superoxide burst activity of neutrophils after PMA stimulation was 0% in NBT assay (controls showed more than 95% burst cells) and 0% cells were oxidized to rhodamine by DHR assay (controls showed more than 95% cells positive to rhodamine) in CGD patients (Table 1). Stimulation index for patients was less in CGD patients (SI in the range of 1–2.2) in comparison to controls (SI in the range of 7.08–96.27). Phenotypic characterization of patients was performed using a flow-cytometry-based approach by use of antibodies directed against components of NADPH oxidase, followed by molecular confirmation (Table 2).

**Table 1:** Basic clinical details and functional parameters recorded for diagnosed CGD subjects.

**Basic clinical parameters**	**Patient 1**	**Patient 2**	**Patient 3**	**Patient 4**	**Patient 5**
Age of diagnosis (months)	26	68	13	18	8
TLC (cells/μl)	9,200	18,540	21,580	10,130	20,710
ANC (cells/μl)	1,490	12,690	5980	2670	9,620
ALC (cells/μl)	740	4,264	12,732	5,768	9,160
Hemoglobin levels (g/dl)	8.2	7.2	10.3	11.1	9.1
Pneumonia (no. of infections)	1	4	2	1	1
Abscesses (no. of infections)	0	2	0	2	1
Site of infection	Right middle lobe	Lung, GI tract, gums	Lung	Perianal, liver, lung,	Right sub-mandibular, lung
Organism isolated	*S. aureus*	*E. coli*	Nil	Nil	*S. aureus*
Failure to thrive	Yes	Yes	No	Yes	No
^*^NBT (%)	0	0	0	0	0
^*^DHR (%)	0	0	0	0	0
DHR assay ^*^stimulation index (SI = S/N)	1	2.2	1.16	1.125	1

* *Respective control samples were also tested along with subjects*.

**Table 2:** Flow cytometric evaluation of expression of NADPH oxidase components in control and patient's neutrophils.

**Patient no**.	**Defective component of NADPH oxidase**	**Signal (S)/noise (N) ratio for component staining**		**Gene involved**
		**S/N ratio for patient**	**S/N ratio for control**	
1	p47^phox^	4.71	11.42	*NCF1*
2	p47^phox^	3.23	5	*NCF1*
3	p47^phox^	3.19	11.11	*NCF1*
4	P22^phox^	1.45^#^	14.46	*CYBB/CYBA*
5	P22^phox^	1.95	72.21	*CYBB/CYBA*

*MFI of stained (S) to unstained (N) neutrophils in patients and controls.

# *Mother showed mosaic pattern after staining with anti-p22^phox^ antibodies indicating patient having defect in X-linked component, i.e., gp91^phox^*.

### Defective NADPH Oxidase Component Expression in CGD Subjects

**Figure 1:**
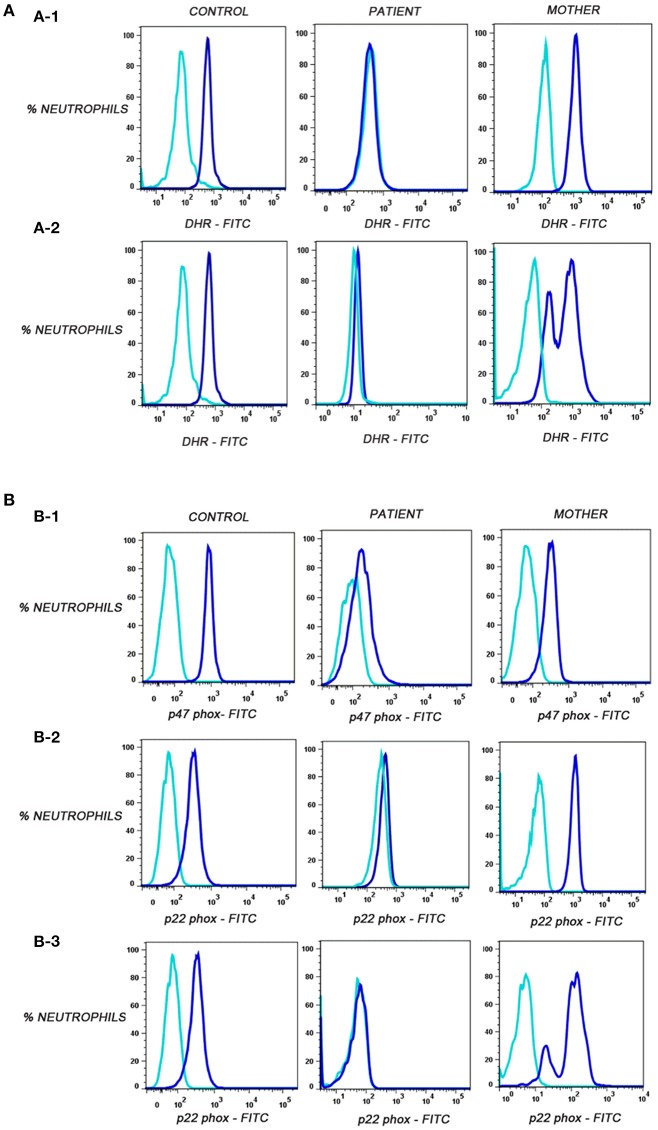
**(A)** Flow cytometric evaluation of dihydrorhodamine-123 (DHR) assay on neutrophils in control, corresponding mother (to distinguish XL-CGD from autosomal CGD patient), and CGD patients. Unstimulated neutrophils (sky blue) showed no oxidation of dihydrorhodamine-123 (DHR) reagent to rhodamine in contrast to stimulated neutrophils (blue) by PMA. **(A-1)** Oxidation of DHR in patient P1 representative of autosomal recessive CGD(P1,P2,P3)/*de novo* X-linked CGD (P5) patient, control, and mother's sample. **(A-2)** Oxidation of DHR in patient P4-X-linked CGD patient, carrier mother, and control sample. **(B)** Flow cytometric evaluation of p47^phox^ and p22^phox^ expression on neutrophils. Median fluorescent intensities were recorded for stained (blue) and unstained (sky blue) neutrophils in control, patient, and mother's sample. **(B-1)** Defective p47phox component expression in patient P1, control, and mother. **(B-2)** Defective p22^phox^ component expression in patient P5, control, and mother*. **(B-3)** Defective p22^phox^ component expression in patient P4, control, and mosaic pattern in mother clearly indicating X-linked defect (gp91^phox^ defect) in patient P4. *Important to further confirm by molecular characterization in patient and parents.

**Figure 2:**
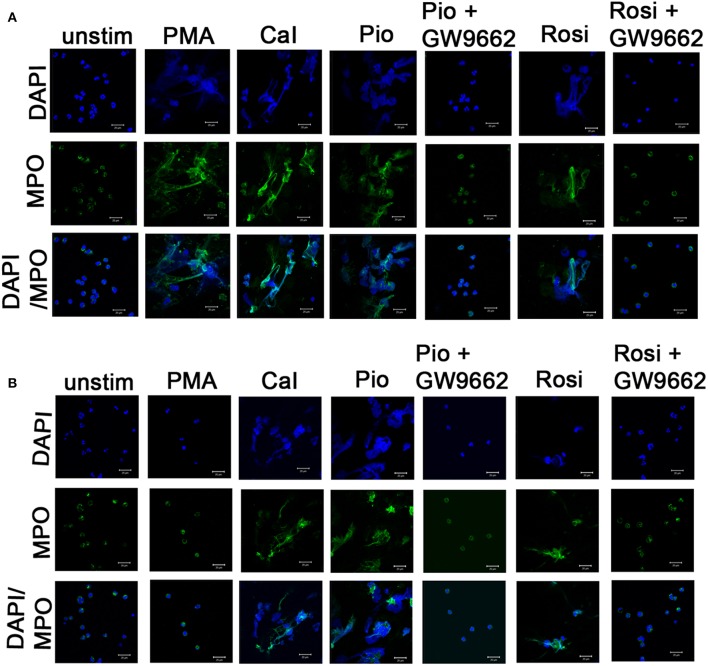
Induction of NETosis by PPARγ agonists. Human neutrophils from one control **(A)** and representative CGD **(B)** patient with homozygous delGT mutation were stimulated with 14 μg/μl of pioglitazone and 15 μg/μl of rosiglitazone for specified time intervals. Cells were treated with or without GW9662 along with PPARγ agonist treatment. Cells were then fixed with 4% PFA and stained with DAPI (nuclear stain) and antibodies against MPO and observed under confocal microscopy (63×). Scale bars, 20 μm. Images are representative of three independent experiments. *NETs formation was observed in a similar way in autosomal recessive and X-linked CGD cases and no difference was observed in both categories. **(B)** Representative image taken from a P2 patient with homozygous delGT.

Out of these five male patients, P4 patient's mother's sample showed carrier mosaic pattern in NBT and DHR (Figure 1A-1) assays, clearly suggesting X-linked (XL) CGD having defect in CYBB gene encoding gp91^phox^ in the patient. In the remaining four patients (P1, P2, P3, and P5), where mother's sample showed normal NBT (95–99% burst cells) and DHR (Figure 1A-2) (95–99% cells showed oxidation to rhodamine), they were further evaluated for NADPH oxidase component expression such as p22^phox^, p47^phox^, and p67^phox^ encoded by genes *CYBA* (cytochrome b-245 alpha subunit), *NCF1* (neutrophil cytosolic factor 1), and *NCF2* (neutrophil cytosolic factor 2). Out of these patients, three out of five (P1, P2, and P3) patients showed defect in expression of p47^phox^ detected by anti-p47^phox^ antibody (Table 2; Figure 1B-1) and one patient (P5) showed defect in expression of p22^phox^ detected by anti-p22^phox^antibody (Table 2; Figure 1B-2). Signal (S)-to-noise (N) ratio for CGD patients was in the range of 1.45 to 4.71 in comparison to control (in the range of 5 to 72.21) for component expression (showing absent or reduced shift of stained cells) in comparison to unstained neutrophils (Figure 1B).

**Figure 3:**
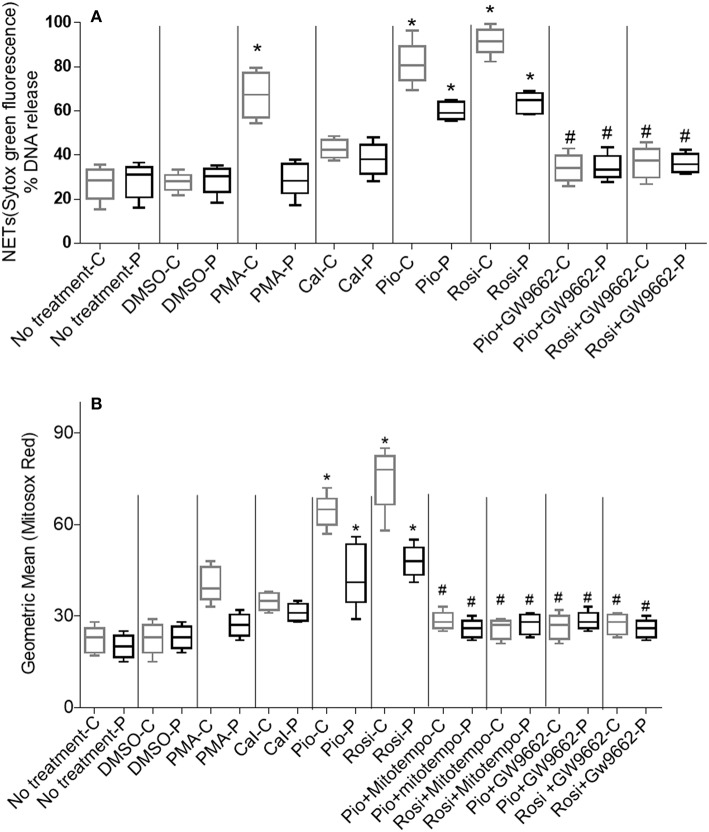
**(A)** Quantitation of NETosis using Sytox green by fluorimetry. Neutrophil extracellular trap (NETs) formation was quantified by fluorimetry after treatment of neutrophils from control and CGD cohort with PPARγ agonists only/PPARγ agonist + antagonist treatment, using 5 μM Sytox green dye. % DNA release was calculated. Graph shows mean ± SD from three independent experiments for each patient. Statistically significant comparisons were obtained by unpaired *t*-tests and comparisons are as follows: *Respective patient/control cohort compared to untreated cells (*p* < 0.0001). ^#^Respective patient/control cohort compared to respective agonists only (*p* < 0.001). -C, controls; -P, patients; PMA, phorbol myristate; Cal, calcium ionophore; Pio, pioglitazone; Rosi, rosiglitazone. **(B)** Quantitation of mitochondrial ROS using MitoSOX red by fluorimetry: PPARγ agonist treatment enhances production of mitochondrial ROS by neutrophils from control and CGD patient neutrophils with or without MitoTempo (Mitochondrial ROS inhibitor)/with or without GW9662 treatment. Mitochondrial ROS was quantified by MitoSOX red and represented as geometric mean. Graph shows mean ± SD from three independent experiments for each subject. *Respective patient/control cohort compared statistically with respective untreated cells of patient/control (*p* < 0.05). ^#^Respective patient/control cohort compared statistically with respective group agonists only (*p* < 0.05).

Interestingly, patient P4 having XL-CGD (mother showing mosaic pattern in NBT and DHR assay) showed abnormal expression of p22^phox^ and mother's sample showed mosaic pattern for p22^phox^ expression using anti-p22^phox^ antibody staining (Figure 1B-3). gp91 and p22^phox^ components together are involved for stable expression of these components on transmembrane/phagosomes of neutrophils. Hence, defect in any one of these (gp91^phox^ and p22^phox^) results in defective expression of p22^phox^ detected by anti-p22^phox^ antibody. Hence, the patient can be diagnosed as XL-CGD if the mother shows mosaic neutrophil burst activity in NBT, DHR, and p22^phox^ expression. However, X-linked defects may also arise from *de novo* mutations in germline cells and will therefore not always be present in the somatic cells of the mother. Hence, failure to define the mother as an X-linked carrier does not disprove the possibility of X-linked CGD status of the patient; hence, further confirmation should be done by molecular characterization. Molecular details for CGD patients are mentioned in Table 3.

**Table 3:** Molecular characterization of patients phenotypically diagnosed as CGD.

**Patient no**.	**Defective protein**	**Defective gene**	**Mutation type**	**Nucleotide change**	**Protein change**	**Location**
1	p47 phox	*NCF1*	Not determined	Not determined	-	-
2	p47 phox	*NCF1*	Deletion	c.75_76delGT	p. Y26HfsX26	Exon 2
3	p47 phox	*NCF1*	Deletion	c.75_76delGT	p. Y26HfsX26	Exon 2
4	gp91phox	*CYBB*	Splice site	c. 338-4T>A	Not applicable	Intron 4
5	gp91 phox	*CYBB*	Missense	c.1546T>A	p.W516R	Exon 12

### Pioglitazone and Rosiglitazone Results in Formation of Neutrophil Extracellular Traps in CGD Subjects

In this study, two representative and well-described NET inducers, PMA and calcium ionomycin, which induce NETosis over a period of 3–4 h, were used. Post-stimulation (after 3 h), neutrophils were fixed with PFA and stained with antibody directed against MPO and DNA was stained with DAPI. Activated neutrophils with both the stimulants individually resulted in a similar NET structure containing extracellular DNA co-localized with MPO (Figures 2A,B). NETs were quantified using Sytox green and control samples had a significantly (*p* > 0.0001) higher rate of NETosis in comparison to CGD patients after PMA stimulation. Calcium ionomycin induced NETosis in both CGD (*p* = 0.2249) and control cohort (*p* = 1.0), indicating phox ROS-independent NETosis formation. The effect of pioglitazone and rosiglitazone was detected on neutrophils to induce neutrophil extracellular traps formation in CGD patients and controls with/without PPARγ antagonist (GW9662). Both fluorimetry (by use of Sytox green dye) and confocal microscopy (by using DAPI and anti-MPO antibody) suggested release of extracellular DNA lattices in both control and CGD patients (Figures 2A,B). Granular staining of MPO, which signifies extracellular release of DNA by means of NETosis, was also observed in both cohorts post-agonist treatment (Figures 2A,B). Neither apoptosis nor necrosis leads to NET formation, as demonstrated by quantifying extracellular DNA (20). NET formation was significantly higher after both pioglitazone and rosiglitazone treatment compared to cells untreated or treated with DMSO alone (Figures 2A,B). Significant neutrophil extracellular trap formation was observed after pioglitazone and rosiglitazone treatment alone in CGD subjects irrespective of germline mutation (*p* < 0.0001; Figure 3A). PPARγ antagonists inhibited NET formation significantly (*p* < 0.001) in CGD cases as well as controls (Figures 2A,B, 3A).

### Enhancement of mtROS Production in Phagocytes Post-pioglitazone and Rosiglitazone Treatment

It is known that pioglitazone had little effect on mitochondrial content in blood phagocytes as detected by mitotracker green (12). We studied mitochondrial ROS production by means of MitoSOX red independent of Mitotracker green. MitoSOX red specifically detects superoxide (ROS) molecules generated by mitochondria. Pioglitazone was previously shown to induce mitochondrial ROS formation in XL-CGD monocytes and phagocytes (12). We found that neutrophil mitochondrial ROS formation was enhanced significantly by both pioglitazone and rosiglitazone (*p* < 0.05) regardless of whether the CGD was due to gp91^phox^ or p47^phox^ deficiency (Figure 3B). Mitochondrial ROS was abrogated (*p* < 0.05) in cells treated with the PPARγ antagonist/mitochondrial ROS inhibitor MitoTempo in both control and CGD neutrophils. No marked variation in mitochondrial ROS generation post-pioglitazone or rosiglitazone treatment was observed in CGD cases with varying germline mutations (Figure 3B), indicating that PPARγ-induced mitochondrial ROS production is independent of the NADPH oxidase.

## Discussion

Due to defective NADPH oxidase enzyme, phagocytes from CGD patients fail to form ROS. ROS is an essential component for formation of NETs that is responsible for clearance of infections by oxidative burst formation. Component defect is a significant marker for residual ROS formation, indicating higher residual ROS production in autosomal recessive patients than X-linked CGD cases (27). ROS production by neutrophils in small amounts confers a significant survival benefit for CGD patients irrespective of NADPH oxidase component defect (27). Hence, attempts are made to increase ROS production in CGD patients for better management (12). In an earlier study, an NADPH-independent ROS producer such as pioglitazone was found to induce mitochondrial ROS production in epithelial cell lines (28). Recently, the PPARγ agonist pioglitazone was found to induce mitochondrial ROS formation in murine X-linked CGD models (12). The effect of both pioglitazone and rosiglitazone in induction of mitochondrial ROS production was significantly high in our CGD subjects. Mitochondrial ROS formation in CGD cases might be beneficial for clearance of recurrent infections as PPARγ agonists play a role in infection clearance in sepsis cases (11).

Mitochondrial ROS is essential for NOX-independent NETosis (23). Mitochondrial oxidative stress was also found to induce NET formation (29). Neutrophil extracellular traps formation is impaired in CGD patients and can be reversed by PPARγ agonist treatment, regardless of the molecular nature of the NADPH oxidase defect. The NETosis rate is significantly higher (*p* > 0.0001) in CGD subjects treated with PPARγ agonists in comparison to untreated cells/antagonist-treated cells. Induction of NETosis by mitochondrial ROS is dependent on various signaling cascades such as AKT and p38 (23). Although exact mechanism for induction of NETosis by mitochondrial ROS production independent of NADPH oxidase is not known, knockdown studies will be helpful in the near future to explore signaling pathways essential for induction of mitochondrial ROS formation after PPARγ agonist treatment.

Excessive ROS formation can cause tissue damage, ultimately leading to inflammation. Site, source, and intensity of ROS regulate macrophage signaling and polarization into M1/M2 (30). M2 macrophages are considered as anti-inflammatory. Mitochondrial ROS also controls regulation of anti-inflammatory phenotype of M2 macrophages via activation of the NF-kB pathway (31). PPARγ gene regulation system regulates activation of the NF-kB pathway. Knockdown studies of enzyme components downstream of the NF-kB pathway will be helpful in exploring the mechanism of mitochondrial ROS activation post-PPARγ agonist treatment. In the future, anti-inflammatory cytokine kinetics pre- and post-pioglitazone treatment should be studied in monocytes of CGD subjects, which will make PPARγ agonist, specifically pioglitazone treatment therapy, more promising. Limitations of this study are *in vitro* detection of gain of mitochondrial ROS/NET-mediated microbicidal activity and repetition of *in vitro* NETosis after intermittent *in vivo* administration of the two drugs in any of these five pediatric patients. Gain of microbicidal activity following NADPH oxidase-independent ROS/NET formation will be studied in the near future. Also, this study was performed in a small number of CGD cases.

Apart from the proposed experimental studies regarding the detailed mechanism of action, controlled trials could provide valuable information regarding the clinical use of pioglitazone in CGD patients. Curative HSCT remains challenging in developing countries for various reasons such as delayed referral/diagnosis, severe infections, lack of experienced transplantation centers, and limited resources (32). Clinical use of pioglitazone as intermittent therapy in a single pediatric CGD case has already been reported (33) and is now being investigated in a human CGD trial^1^.

## Data Availability

The datasets generated for this study are available on request to the corresponding author.

## Ethics Statement

The study was conducted under Ethics approval from Bai Jerbai Wadia hospital for children (reference number: IEC-BJWHC/AP/2017/012).

## Author Contributions

GH performed all the laboratory assays, data analysis, and manuscript writing. UB helped in data analysis and reviewed the manuscript. MK and PK performed molecular analysis of the study. PT helped in performing laboratory investigations. MD and MM supervised and reviewed the study.

### Conflict of Interest Statement

The authors declare that the research was conducted in the absence of any commercial or financial relationships that could be construed as a potential conflict of interest.

## Footnotes

^1^https://clinicaltrials.gov/ct2/show/NCT03080480
